# Rare Case of Four Osseous Lesions of the Skull in a Patient with Secondary Syphilis

**DOI:** 10.1155/2018/3148758

**Published:** 2018-05-08

**Authors:** Jace Kusler, Supha Arthurs

**Affiliations:** ^1^University of North Dakota School of Medicine and Health Sciences, 1301 N. Columbia Rd., Grand Forks, ND 58203, USA; ^2^CHI St. Alexius Health, 900 E. Broadway Ave., Bismarck, ND 58503, USA

## Abstract

Syphilis is a sexually transmitted infection that is caused by the bacterium *Treponema pallidum.* Syphilis can present as primary, secondary, tertiary, or congenital. It can have an effect on many different organ systems and tissues leading to a wide variety of symptoms and complications; one rare manifestation is bone involvement. The patient in this case was diagnosed with secondary and early neurosyphilis and was also found to have skull lesions that were due to *Treponema pallidum*. There are guidelines for the treatment of primary, secondary, tertiary, and congenital syphilis; however, there are currently no guidelines for the treatment of syphilis with bone involvement.

## 1. Introduction

Bone involvement is an unusual manifestation of syphilis, which is caused by the bacterium *Treponema pallidum*. Although there are not currently guidelines for treatment, the literature has shown treatment with high-dose penicillin therapy to have favorable outcomes. This report details a case of secondary and early neurosyphilis with four osseous lesions of the skull. Of particular significance, this case demonstrates the rare finding of osseous lesions with secondary syphilis and successful treatment with 14 days of 24 million units per day of IV penicillin G.

## 2. Case Report

**Figure 1:**
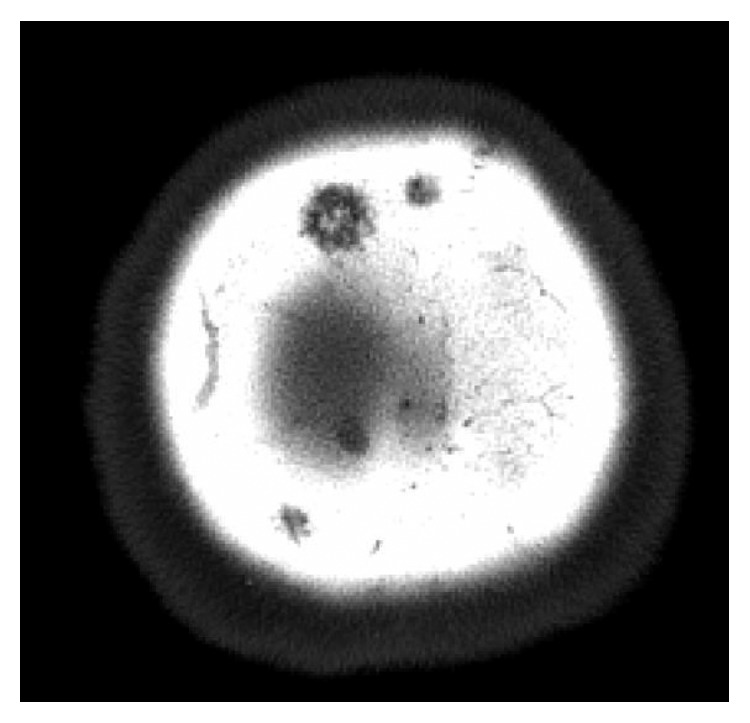
Lesion of right parietal bone.

**Figure 2:**
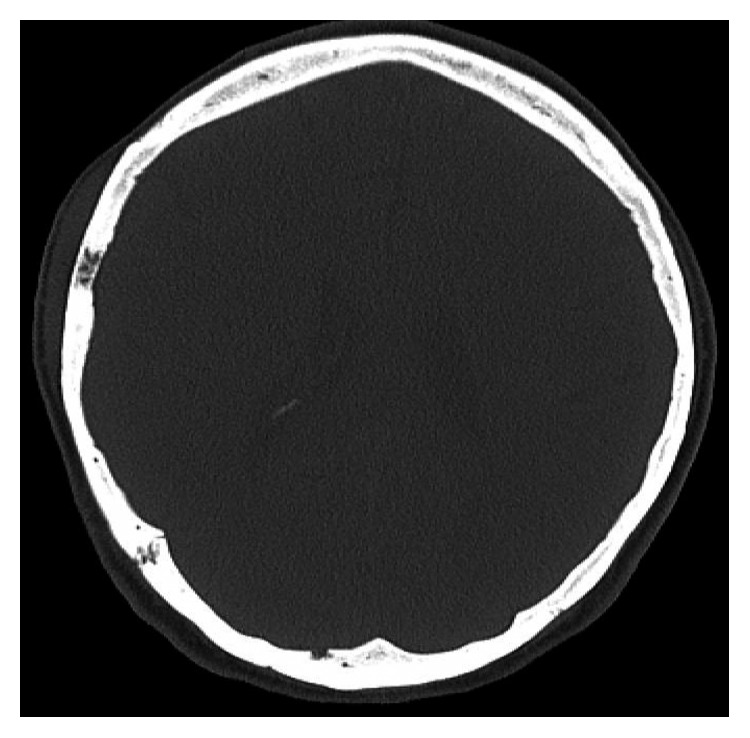
Lesion of right temporal bone.

**Figure 3:**
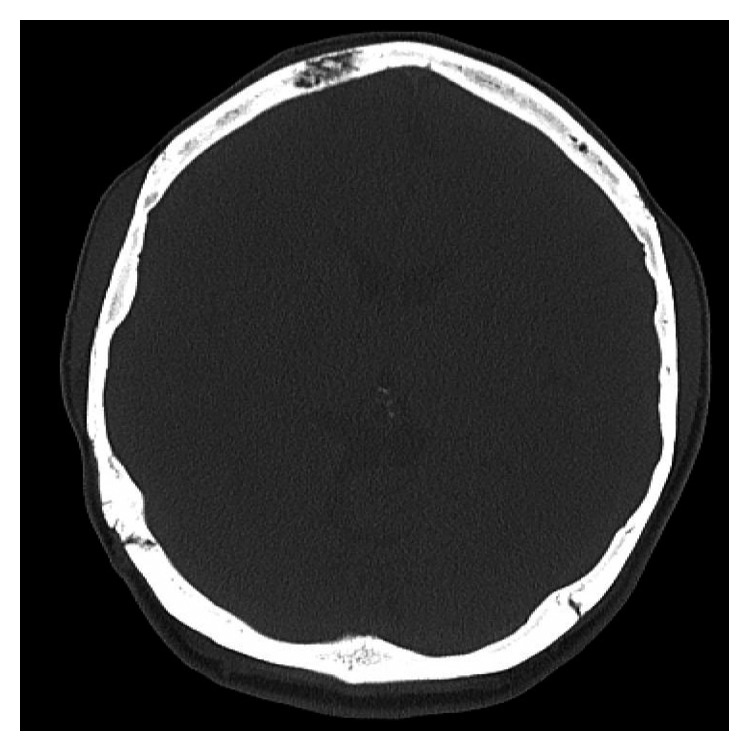
Lesion of right frontal bone.

**Figure 4:**
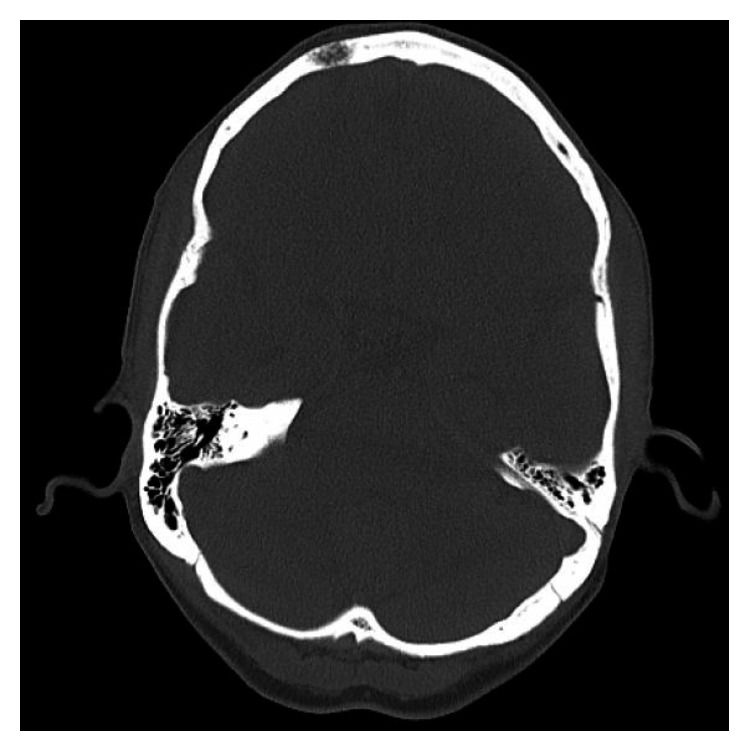
Lesion of right frontal bone.

A 50-year-old Native American female presented to the ER with a bladder infection, and a urine culture grew *E. coli*. She was treated with trimethoprim/sulfamethoxazole and developed a diffuse rash including the palms and soles. There was no lymphadenopathy present upon physical exam. One month later, she presented to the clinic with a month of severe pounding headaches, blurry vision in the right eye, and pain over the temporal area on the right side. Her ESR was elevated with a normal CRP. The suspected diagnosis was giant cell arteritis, and she was started on 60 mg of oral prednisone daily that helped the headaches. A temporal artery biopsy was done and showed no evidence of vasculitis. The patient was tapered from the steroid and started having progressive blurry vision and floaters. Three months later, the patient presented to the emergency department and was admitted to the hospital due to the blurry vision and floaters at which time she had a positive fluorescent treponemal antibody absorption test. She was HIV negative. The patient had a Venereal Disease Research Laboratory test of the cerebral spinal fluid done which came back negative. Her cerebral spinal fluid cell count was 178 nucleated cells with 164 lymphocytes, and protein was 53 mg/dl. She had a serum rapid plasma reagin titer of 1 : 1024. She was diagnosed with secondary and early neurosyphilis for which she was started on 24 million units per day of IV penicillin G, and severe uveitits for which she was started on 60 mg of oral prednisone and 1% prednisolone acetate eye drops. The uveitis was likely secondary to the syphilis infection. A CT scan of the head showed four osseous lesions (Figures 1–4). An immunohistochemical stain of a skull biopsy was done which came back positive for *Treponema pallidum*, thus confirming syphilis as the cause of the bone lesions. An echocardiogram was performed which did not show any significant cardiac defect. The patient completed 14 days of 24 million units per day of IV penicillin G, and there was no evidence of a Jarish–Hexheimer reaction. Upon follow-up, the patient still complained of blurry motion, but denied a headache or skin rash. The eye exam showed improvement and showed no signs of ocular syphilis. Four months after discharge from the hospital, her blood rapid plasma reagin titer was 1 : 256 that is more than a fourfold decrease from her baseline, and a CT scan of the head showed the osseous lesions were unchanged from the previous CT scan. Three months after her previous follow-up, her serum rapid plasma reagin titer was 1 : 128. A repeat lumbar puncture showed 18 nucleated cells with 16 lymphocytes, and protein was 42 mg/dl. A repeat Venereal disease research laboratory test of the cerebral spinal fluid was done and came back positive with a 1 : 8 titer that was negative on the previous exam, which is hypothesized to be a false negative result. The patient was lost to further follow-up.

## 3. Discussion

Bones can be affected at all stages of syphilis, but it is more common in tertiary and congenital infection [1]. Cases of syphilis with bone involvement have been reported in patients who are HIV positive as well as patients who are HIV negative [2]. Bone lesions can occur without any clinical signs or symptoms; however, the most common presentation is bone pain [2–4]. In some cases when skull lesions are present, patients report headaches [1].

The long bones and skull are most commonly affected by syphilis; however, there have been cases affecting the ribs, clavicle, spine, and sternum [2]. Bone lesions can be detected with plain radiography, CT scan, MRI, or bone scintigraphy [5]. Identification of spirochetes in bone lesions with dark-field microscopy, silver stain, immunoperoxidase stain, or polymerase chain reaction is uncommon and occurs even less in late-stage disease [2, 5].

The current guidelines for treatment of syphilis is parenteral penicillin G. Patients with primary, secondary, or early latent syphilis are to be treated with a single intramuscular dose of 2.4 million units of benzathine penicillin G. Patients with tertiary and late latent syphilis are to be treated with 7.2 million total units of benzathine penicillin G, and patients with neurosyphilis are to be treated with 18–24 million units per day for 10–14 days of aqueous crystalline penicillin G [6]. There are currently no treatment guidelines for syphilis with bone lesions; however, 2.4 million units of intramuscular benzathine penicillin G for two to four weeks has been shown to be an effective treatment [1–3]. Symptomatic relief occurs rapidly after the start of therapy. After completion of appropriate therapy, there can be complete radiographic resolution of the lesions; however, the osseous lesions can persist for 7–11 months [5, 7].

Although bone involvement is a rare complication of syphilis, it is important to include syphilis in the differential diagnosis as a cause of bone pain or radiographic osseous lesions. There are currently no treatment guidelines for syphilis with bone lesions; however, there have been cases that have shown successful treatment with 2.4 million units of intramuscular benzathine penicillin G for two to four weeks. In this case, 14 days of 24 million units per day of IV penicillin G was given due to early neurosyphilis, and this treatment was effective in resolving the patient's bone pain and reducing her rapid plasma reagin titer by fourfold; however, the patient was lost to follow-up and thus follow-up imaging was not obtained.
